# Understanding the genetic determinant of severity in viral diseases: a case of SARS-Cov-2 infection

**DOI:** 10.1186/s43042-020-00122-z

**Published:** 2020-12-31

**Authors:** Babayemi Olawale Oladejo, Covenant Femi Adeboboye, Tinuola Tokunbo Adebolu

**Affiliations:** Department of Microbiology, Federal University of Technology, P.M.B. 704, Akure, Nigeria

**Keywords:** Coronavirus, COVID-19, SARS-CoV-2, Human leucocyte antigen (HLA) allele, Toll-like receptor (TLR), Angiotensin-converting enzyme 2 (ACE2) gene

## Abstract

**Background:**

Numerous research studies have identified specific human gene variants that affect enhanced susceptibility to viral infections. More recently is the current pandemic where the SARS-CoV-2 infection has shown a high degree of person-to-person clinical variability. A wide range of disease severity occurs in the patients’ experiences, from asymptomatic cases, mild infections to serious life threatening conditions requiring admission into the intensive care unit (ICU).

**Main body of the abstract:**

Although, it is generally reported that age and co-morbidities contribute significantly to the variations in the clinical outcome of the scourge of COVID-19, a hypothetical question of the possibility of genetic involvement in the susceptibility and severity of the disease arose when some unique severe outcomes were seen among young patients with no co-morbidity. The role human genetics play in clinical response to the viral infections is scarcely understood; however, several ongoing researches all around the world are currently focusing on possible genetic factors. This review reports the possible genetic factors that have been widely studied in defining the severity of viral infections using SARS-CoV-2 as a case study. These involve the possible involvements of ACE2, HLA, and TLR genes such as TLR7 and TLR3 in the presentation of a more severe condition.

**Short conclusion:**

Understanding these variations could help to inform efforts to identify people at increased risk of infection outbreaks through genetic diagnosis of infections by locating disease genes or mutations that predispose patients to severe infection. This will also suggest specific targets for therapy and prophylaxis.

## Background

The world still battles one of the greatest health threats of the century, a global pandemic. As described by the World Health Organization (WHO), the disease which is named COVID-19 is caused by a new coronavirus referred to as severe acute respiratory syndrome coronavirus-2 (SARS-CoV-2) [1, 2]. This virus shows some significant differences from the severe acute respiratory syndrome coronavirus (SARS-CoV-1), which was first identified in 2003, and the Middle East respiratory syndrome corona virus (MERS-CoV) [2, 3]. Although the pathogenesis of the SARS-CoV-1 and MERS-CoV has been sufficiently understood, however, there are still a lot of unanswered questions concerning the pathogenesis of the SARS-COV-2 virus. The spread of the virus has affected all the regions of the world. According to the WHO report (10 November 2020), the global number of cases of COVID-19 has significantly increased, totaling more than 3.6 million new cases, while new deaths have increased to over 54,000. This brings the cumulative numbers to over 49.7 million reported cases and over 1.2 million deaths globally since the start of the pandemic [4]. The disease has also contributed significantly to the global disease burden, creating a devastating effect on the world economy. The SARS-CoV-2 spreads through person-to-person contact through respiratory droplets produced when an infected person coughs or sneezes within a proximity to an uninfected individual majorly at a distance of about 6 ft from each other. Significantly, another way of spreading the virus is by touching the mouth, nose, or eyes after contact with various surfaces or object with the virus [1]. Clinical manifestations of the infection include fever, fatigue, non-productive cough, decreased leucocyte counts, and radiographic evidence of pneumonia which are very similar to the clinical symptoms of SARS-CoV and MERS-CoV [5]. Among the several confounding aspects of this novel coronavirus is that a wide range of disease severity occurs in patients’ experiences. Although only a minority of COVID-19 patients would eventually require hospitalization, yet the effects of the infection for these classes of people are in some cases life threatening. General observations suggest that the SARS-CoV-2 causes severe symptoms mainly in aged patients, most especially those with underlying chronic disease conditions. However, it becomes more disturbing when two different cases of a pair of previously healthy young brothers with an average age of 26 years from two different families require admission to an intensive care unit (ICU) in rapid succession, as was the reported case at Radboud University Medical Center, Nijmegen, Netherlands [6]. Another scenario is when health care workers that have been severally exposed to the virus remain healthy and uninfected while some become infected and die. These unusual cases raise further questions on the consideration of genetic factors and their possible role in compromising the immune system of the four young men and even presupposed resistance to the virus among some patients.

Generally, viral infections have shown certain inter-individual clinical variability. Genetic variation among humans produces a wide variety of responses to viral infections. For instance, in an early genetic examination of poliovirus infection among twins, at least one twin was diagnosed with paralytic poliomyelitis in the study [7]. The probability of paralytic poliomyelitis in the second twin was significantly increased among identical twins as against fraternal twins. This indicates a genetic influence on the outcome of the infection. Below are some examples of previously studied genetically mediated susceptibility and severity of viral infections (Table 1).

**Table 1:** Some genetic variants associated with particular disease outcomes in specific viral infections

Viral infections (condition)	Associated gene variants	Disease outcome
Influenza virus (severe pneumonitis)	*IRF7*, *IRF9*, *TLR3*, *IFITM3*, *SFPA/B*	Susceptibility [7–9]
Rhinovirus (severe pneumonitis)	*IFN1*	Susceptibility [7]
Human immunodeficiency virus	*CCR5*, *HLAB57*	Resistance [7, 10, 11]
Herpes simplex virus (encephalitis)	*TLR3*, *TRIF*, *TRAF3*, *IRF3*, *TBK1*	Susceptibility [7, 12, 13]
Norovirus and rotavirus	*FUT2*	Resistance [7, 14, 15]
Respiratory syncytial virus (bronchiolitis)	*IL4*, *IL4RA*, *IL8*, *IL10*, *IL13*, *SFPA/D*	Susceptibility [7, 16–18]

The reason why some people get severe and life-threatening COVID-19, while others are completely asymptomatic or suffer just mild symptoms is worth digging extensively into, most especially with the world searching for answers through research for both therapeutic and prophylactic measures against the virus [19].

## Pathogenesis of SARS-CoV-2 virus

The first stage of the SARS-CoV-2 is usually an asymptomatic state with an initial of 1–2 days of the infection, and it is initiated when the inhaled virus SARS-CoV-2 likely binds to epithelial cells in the nasal cavity and starts replicating. Angiotensin-converting enzyme 2 (ACE2) is the main receptor for both SARS-CoV-1 and 2 [20]. In SARS-CoV-2 infection, cell entry is facilitated by the ACE2 which functions together with trans-membrane serine protease 2 (TMPRSS2). ACE2 is an important enzyme produced in the renin–angiotensin system (RAS) for a counterbalance action. The propagated virus migrates down the respiratory tract along the conducting airways and leads to a more robust innate immune response being triggered possibly through the activation of divers Toll-like receptors (TLRs) [20]. At this time, the disease, COVID-19, becomes clinically manifested. The level of Cys-X-Cys (C-X-C) motif chemokine ligand 10 (CXCL10) and some other innate response cytokine such as interferon (IFN)-alpha, IFN-gamma, interleukin (IL)-1β, IL-6, IL-12, and tumor necrosis factor (TNF)-alpha may be predictive of the subsequent clinical course [6]. Epithelial cells that are infected with the virus are a major source of both beta and lambda interferon [20]. CXCL10 is an interferon-responsive gene that has an excellent signal to the alveolar type II cell response to both SARS-CoV and influenza [21, 22]. CXCL10 has also been reported to be useful as a disease marker in SARS [20, 23]. In the final stage, hypoxia and progression to acute respiratory distress syndrome (ARDS) are usually seen, characterized by cases of development of pulmonary infiltrates and very severe disease [20]. Acute respiratory distress syndrome (ARDS) is the main cause of death in most COVID-19 patients. An early survey of 41 SARS-CoV-2-infected patients in admission during the outbreak showed that six of them died from ARDS [24]. ARDS is majorly experienced as shortness of breath, and it is a common immune pathological event in SARS-CoV and MERS-CoV infections [25]. Several other literatures revealed that a vital mechanism for development of ARDS is cytokine storm [26, 27]. Cytokine storm arises from the release of large numbers of pro-inflammatory cytokines such as IFN-alpha, IFN-gamma, IL-1β, IL-6, IL-12, and TNF-alpha and also some chemokines like CCL2, CCL3, CCL5, CXCL8, CXCL9, and CXCL10 by immune effector cells in SARS-CoV-2 infection [6]. This cytokine storm triggers a deleterious attack by the immune system on the body, which in the case of COVID-19 is on the healthy lung tissues of patients. The pathological result of SARS-CoV and COVID-19 is diffuse alveolar damage with fibrin-rich hyaline membranes and a few multinucleated giant cells [20]. Individuals at older ages are particularly at higher risk because of their diminished immune response and inefficient ability to repair the damaged epithelium. The elderly also have reduced mucociliary clearance, and this may allow the virus to spread to the gas exchange units of the lung more readily [28].

## Genetic perspective to the determinants of severity of viral infections and the COVID-19

Genomics generally plays a crucial role in the genetic landscaping and generation of information about susceptibility, severity, and protection against infectious diseases [29]. Host genes have been shown to influence the severity of several infections, viral replication, and inflammation among various distinct individuals. Considering the possible implications of these host genes in the entry, replication of viruses, the processes that are involved in mounting host immune response and development of inflammation, etc., it appears that either single genes or multiple genes might be crucially involved in the processes. Genes that affect susceptibility and severity of viral diseases may be classified functionally into categories like virus entry receptors, co-receptors, or receptor-modifying enzymes. Also, gene polymorphisms affecting the expression on protein production of specific cytokines can influence viral disease severity [7, 20]. Genetic mutations or defects in various other aspects of cellular innate and adaptive immune responses to viral infections, such as signaling response to viruses, activity of antiviral restriction factors, or proper initiation of T cell responses, have also been associated with enhanced severity of numerous viral infections. Identification and further research on such classifications can provide a backbone for identifying novel biologically plausible susceptibility or resistance gene loci [7]. These classifications may likely increase as discovery of potential new genetic determinants of viral diseases continue. Researchers have adopted several approaches to identify genetic factors that are linked to virus infection susceptibility or disease outcome, many of which involves the identification of individuals with unusual responses to viral infections, such as abnormalities in the severity of illness, or rare complications. The patient’s genes can be studied [7, 20]. This has proven successful for identifying disease susceptibility and severity loci among such individuals. Many of the studies that have been carried out have focused on immunity-related genes, such as human leukocyte antigen (HLA) genes or genes associated with effector function of antiviral interferons (IFNs). For many viral infections, specific HLA alleles have been implicated in susceptibility and severe infection due to the ability of distinct HLA variants to present unique peptide repertoires to T cells [7]. In the case of SARS-CoV-2 virus, Casanova et al. [29] describes the possibility of some inborn errors of immunity which could be either monogenic (single gene) or Mendelean, and that previously healthy, young patients with severe COVID-19 may carry causal genetic variants. This hypothesis could follow a long line of classical genetic studies since 1905, relating to diverse infections in both plants and animals, including humans; however, they are yet to be supported by reports of specific genetic epidemiological studies in the case of COVID-19 [30], although there are several ongoing researches in this regard. Since there is a dearth of information on the human genetic determinants of susceptibility to other coronaviruses, influenza could likely provide the best comparison. The threshold levels of type I and/or III IFN for protection against SARS-CoV-2 could be similar to the 1918 influenza virus. Interferon (IFN) production serves as a major control mechanism of the immune system in clearing the SARS-CoV-2. IFN-dependent control of the virus could be profoundly impaired during initial infection in patients with early-onset of pneumonia, whereas those whose condition deteriorates later could have milder IFN deficiency or genetically determined excessive inflammation [30]. Another possible genetic determinant of susceptibility and severity in the cases of viral infection proposed by several studies is the variation which exist among the human leukocyte antigen alleles [31–33]. Therefore, considering the functional classifications earlier stated, variation within the angiotensin-converting enzyme 2 (ACE2) gene, alteration of genes regulating the Toll-like receptor, and genetic variability across the three major histocompatibility complex (MHC) class I genes (human leukocyte antigen A [HLA-A], HLA-B, and HLA-C genes) could serve as important genetic gateways to the severity of COVID-19 infection [33]. Availability of genome-wide association studies (GWASs) has been used to identify potential susceptibility genes among cohorts afflicted with certain specific clinical manifestations of viral infections. More recently, whole exome sequencing (WES) has been adopted to identify gene polymorphisms responsible for specific viral disease phenotype [7, 20, 29]. This provides a unique opportunity for accessing these genetic gateways and other genes that could potentially be involved in deficiency of host immunity or susceptibility to viral infections. Furthermore, understanding such genetic basis of severity to viral infections could project the world further into genetic diagnosis of diseases and infections.

## Variations of angiotensin-converting enzyme 2 (ACE2) gene as important gateway for severity of SARS-Cov-2 infection

**Fig. 1:**
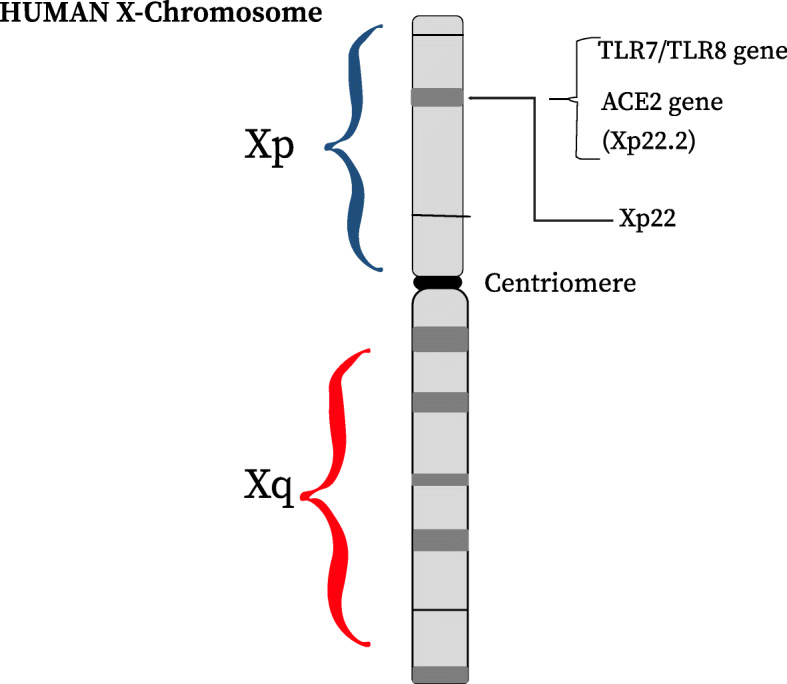
Schematic view of the gene locus of TLR7 and its more similar homolog TLR8 together with the ACE2 gene all present at the Xp22 region on the human X chromosome

The variations within ACE2 genes may be responsible for the dynamics of spatial transmission of COVID-19. The X chromosome also contains a high number of immune-related genes which are responsible for both innate and adaptive immune responses to infection. The ACE2 gene lies on the X chromosome and is found on the locus Xp22.2 (Fig. 1). It is 41.04 kb long and contains 18 or 19 exons which exist in two isoforms (Genbank, NT011757) [34]. Over expression of ACE2 might predispose patients to varying degree of severity of this infection as a result of its potential functional variations which have been shown to alter its activities during transcription [35]. ACE and ACE2 function together in the renin–angiotensin system (RAS) to balance the local vasoconstrictor/proliferative and vasodilator/anti-proliferative actions, resulting in the protection of organs and blood vessels by anti-inflammatory, anti-coagulant, anti-fibrosis, anti-alveolar epithelial cell apoptosis, and anti-oxidative stress activities [33, 34]. Therefore, it is possible that the co-existence of common gene polymorphisms in the ACE and ACE2 genes that alter their mutual expression levels could lead to increased coagulation, capillary permeability, apoptosis in the alveolar cells, accelerated lung damage and pulmonary shutdown which is triggered or complicated by the SARS-CoV-2 infection [34]. Such possibility could either be inherited or mutated. Furthermore, some single nucleotide polymorphisms (SNPs) which are located in the coding regions of the ACE2 gene may also exhibit variations in the allele frequencies among different populations. SNPs like the alternate allele of rs763395248 SNP in T92I risk variant were shown to be relatively higher among individuals of European descent when compared to a global average, comprising populations of individuals of Asian and African descent. This stands in contrast to SNPs like rs758278442 and rs759134032 which are found in the region of protective variants (K31R and Y83H) of ACE2 gene, which show a relatively higher frequency of mutant alleles in Asian populations in comparison to the global average (comprising of populations mostly from European and American descent) [33]. FUT 2 gene variant, a virus entry receptor-modifying enzyme, has also been shown to be genetically involved in the resistance to Norovirus and Rotavirus [7]. Most recently, Cao et al. [35] identified genetic variants via analysis of expression quantitative trait loci (eQTLs) of ACE, which may potentially alter ACE2 gene expression. The analysis was carried out for their frequencies in different populations globally (Table 2). Although it is important to note that while there is a possibility of genetic involvements in the mutual expression levels of ACE and ACE2, scientist have also proposed that the possible cause of these expressions could be based on individual base line health, which further determines which of the two most important angiotensin is expressed in the individual, either angiotensin II or angiotensin 1-7 as seen in the RAS pathway. Young, healthy, and physically fit individual will tend to express more of angiotensin 1-7 which allows for regulation of the pathway and cleavage of ACE2 by enzyme sheddase, leading to less expressions of the ACE2 receptor required for the entry of SAR-CoV-2, unlike in older individuals who have higher risks of developing hypertension, diabetes, heart failure etc., which activates the production of more angiotensin II necessary for higher expression of ACE2. Pathological alterations of the ACE2 pathway seems to cause an increased severity of COVID-19 among patients who suffer from hypertension and diabetes mellitus (DM) as these diseases are both modulated by ACE2 [33]. Several other studies have suggested that males are more susceptible to SARS-CoV-2 infection due to the higher ACE2 density in the lungs. A recent single-cell RNA sequencing (RNA-seq) analysis indicated that Asian males may have a higher expression of tissue ACE2 [13]. This could be due to the presence of ACE2 gene on the X chromosome, with men having only one allele and two in women. Furthermore, estrogen levels have been reported to upregulate ACE2 expression and activity. This creates a double advantage (i.e., two alleles and estrogen upregulation) which may be responsible for the less severity of COVID-19 in females. A report by the Chinese Centre for Disease Control and Prevention (CDCC) showed an overall female mortality of 1.7% of both suspected cases as well as serologically confirmed cases versus 2.8% in males. In serologically confirmed cases, there was a more marked difference of 2.8% female as against 4.7% male mortality [39]. In another study to mark gender differences in this disease, Mehra et al. [40] reported 40% female patients in 8910 COVID-19 patients requiring hospital admission and went on to describe improved survival in female patients, which was independent of older age. These gender-based differences in susceptibility and severity may also be substantially driven by a greater rate of smoking which leads to a higher level of ACE2 expression in the lungs, although no epidemiological study for smoking in the cases COVID-19 were provided [39]. Based on this, it can be assumed that ACE2 may be crucial to the outcomes of COVID-19. Other genes from the RAS-pathway might directly or indirectly influence the balance of ACE1/ACE2 by affecting its main actors (e.g., ABO locus, AGT, SRY, SOX3, ADAM17 AGTR1, and AGRT2) [34]. A limitation to this is that the distribution pattern and influence on differential susceptibility to SARS-CoV-2 infection as well as the genetic basis of differences in expression and functional implications among various patients are still inadequately known.

**Table 2:** Summary of some genetic variants and SNPs associated with viral infections

Classification	Viruses	Important viral genetic variants and SNPs
Virus entry receptor modifying enzyme	Norovirus and rotavirus	FUT2 [7, 14, 15]
	SARS-CoV-2	ACE2 variants (*Lys26Arg*, *Ile468Val*, *Ala627Val*, *Asn638Ser*, *Ser692Pro*, *Asn720Asp*, *Leu731Ile/Leu731Phe*) [35] ACE2 SNPs (*rs4646127*, *rs2158082*, *rs5936011*, *rs6629110*, *rs4830983*, *rs5936029*) [35]
Innate signaling response	Influenza virus	*TLR3*, *IRF7*, *IRF9*, *IFITM3* [7–9]
	Herpes simplex virus	*TLR3, TRIF, TRAF3, IRF3* [7, 12, 13]
	Hepatitis C virus	*TLR7IVS2-151G, TLR8-129G* [36]
	SARS-CoV-2	*TLR3*, *IRF7*, *TLR7* [6, 37]
MHC/HLA Class 1	HIV	*HLA-B57*, *CCR5* [7, 10, 11]
	Dengue virus	*HLA-A 0207* and *HLA-B 51* [30, 33]
	SARS-CoV-1	*HLA-B 4601*, *HLA-B 5401* [32]
	SARS-CoV-2	*HLA-B 1503* [32]
MHC/HLA Class 2	Hepatitis C virus	*HLA-DRB1 0301*, *DQB1 0301*, *DRB1 0101*, *DRB1 0401* [49–56]
	SARS-CoV-2	*HLA-DR* [38]

## Alteration in the toll-like receptor expression (TLR) genes and severity of SARS-Cov-2 infection

The genes responsible for the regulation of Toll-like receptor and the subsequent development of cytokine storm-induced exaggerated inflammatory pathways have been recorded to play a significant role in the severity of COVID-19 and other viral infections [6, 33, 41]. In a complex interaction of the virus and the immune system, the innate immune system recognizes the pathogen-associated molecular patterns (PAMP) expressed by the virus. This causes a ligand-binding activation of the pattern recognition receptor (PRRs) such as the Toll-like receptor, which leads to the expression of specific pro-inflammatory cytokines [41]. The recognition of viral pathogens by the innate immune system is mediated by receptors from two classes of intracellular PRRs: the RLR family and several members of TLRs that recognize nucleic acids. TLR7 and TLR8 recognize single-stranded RNA, while TLR3 recognizes double-stranded RNA [42]. In a study carried out by Wang et al. [36] to describe the associations between TLR7 and TLR8 gene SNPs and susceptibility to hepatitis C virus (HCV) infection, the results suggested that TLR7IVS2-151G and TLR8-129G alleles were present at higher frequency in males of an HCV-infected group as compared to the control group. Based on their ability to recognize single-stranded viral RNA, this suggests that TLR7 and TLR8 play significant role in anti-HCV immune responses [36]. Generally, lung epithelial cells are reported to express all known human TLRs, which include the described expression of TLR3, TLR7, and TLR8 by these epithelial cells [43–45]. Since TLR7 recognizes the single-stranded viral RNA, it is therefore possibly implicated in the disease progression and consequent clearance of SARS-CoV-2 [45]. However, TLR 3 and epithelial lung cells have also been reported as good inducers of the innate signaling pathway by the SARs-CoV-2 virus [37]. Type I interferons (IFN), i.e., (IFNα/β) and type III IFNs (IFN-λ), which can also activate hundreds of antiviral proteins have been shown to be an important cytokine that mediates intracellular clearance of viruses generally and the SARS-CoV-2 as well [7, 20]. Alterations in the genes of the toll-like receptor responsible for the expression of such cytokine could potentially lead to a higher degree of severity of the COVID-19 among patients. For instance, in the case of the two separate families described earlier on, when all genes of the first two brothers were sequenced in search for possible similar cause of increased morbidity, Caspar et al. [6] studied the genes that play significant role in the immune system. This is based on the fact that several of these genes are located on the X chromosome and with the two patients being male, then X-chromosomal gene became most suspicious, since women carry two X chromosomes while men possess a Y chromosome and only one copy of the X chromosome. In any case where men have a defect in such gene, there is no second gene that can take over that role, as in women [6]. This also falls in line with many other reports which proposes that the severity of the SARS-CoV-2 may be linked with the X chromosome, and therefore, men are likely more susceptible. The result of this exome study showed mutations in the gene encoding for the Toll-like receptor 7 (TLR7). A few letters were missing in the genetic code of the TLR7 genes, and as a result, the code cannot be properly read, and almost no TLR7 protein was eventually produced. Impairment in the TLR7 gene could allow the virus to replicate freely since the immune system did not get a message of viral invasion which could have led to activation of the immune signaling cascade that would have produced interferons for the clearance of the SARS-CoV-2 virus [6]. Furthermore, additional confirmation followed when the second pair of brothers who were both under age 35 fell seriously ill with COVID-19, and both of them were also admitted into the ICU for mechanical ventilation. Another investigation on the genetic code of these two brothers via the rapid clinical exome method showed no deletion of genetic codes, but a single spelling mistake of one DNA-letter of the TLR7 gene was identified. However, the resulting effect on the gene was still the same with the previous brothers because they did not make sufficient functional TLR7 protein. This gave a total of four young people with a defect in the same gene, all of whom suffered a highly severe form of COVID-19 [6]. Several genes involved in inflammation are located on the X chromosome. The TLR7 gene is located on the X chromosome on the locus Xp22 (Fig. 1). In the innate immune response to the previous SARS-CoV, TLR3, TLR4, and TLR7 have so far been implicated. The special GU-rich sequences found in the SARS-CoV genome activate TLR7 [7]. TLR3 and TLR4 activate the adaptor TIR-domain-containing adapter-inducing interferon-β (TRIF), whereas myeloid differentiation primary response 88 (MyD88) is the adaptor used by all other TLRs [20]. In the case of more related viral infections like influenza virus pneumonitis and herpes simplex virus encephalitis, severity is associated with defects in the TLR3 pathway [20]. A caveat, however, is that interpretation of data related to mutations or variations in these TLR genes should be carefully studied and managed with caution. This is as a result of the fact that patients recruited for such studies would have to be exposed to other viral infections along life without developing other important RNA-viral infections to ascertain their unique implication for a particular viral infection such as the case of the SARs-CoV-2. Furthermore, polymorphisms in IFN-related genes were reported to affect influenza virus infection outcomes. In a study utilizing WES for a 7-year-old French girl with a severe infection during the 2009 pandemic H1N1 virus, distinct rare mutations in each copy of the patient’s IFN regulatory factor 7 (1RF7) genes were identified. This gene encodes a critical transcription factor involved in type I IFN production [7, 20]. Amazingly, both mutations decreased IRF7 protein function, and cells from this patient allowed uncontrolled high virus replication that was only rescued by treatment with IFN [7]. It is also possible that some inborn errors of immunity or genetic mutations associated with the other TLR genes (TLR 1-10) present in humans which have been shown to be crucial in the activation of the signaling cascade for the production of inflammatory cytokines and the eventual control of intracellular viral multiplication are of significance to the determination of severity among COVID-19 patients [24]. Carter-Timofte et al. [20] suggested that genes which play important roles in inflammation and immunopathology of severe COVID-19 could be further investigated. Such genes are NLRP1, NLRP3, CASP1, MEFV, and several others which all encode proteins involved in inflammasome activation [20]. Others include known genetic causes of hemophagocytic lymphohistiocytosis (HLH) such as defects in the genes PRF1, UNC13D, STX11, STXBP2, LYST, and RAB27A [20].

## Genetic variation of human leucocyte antigen (HLA) genes in severity of SARS-Cov-2 infection

**Fig. 2:**
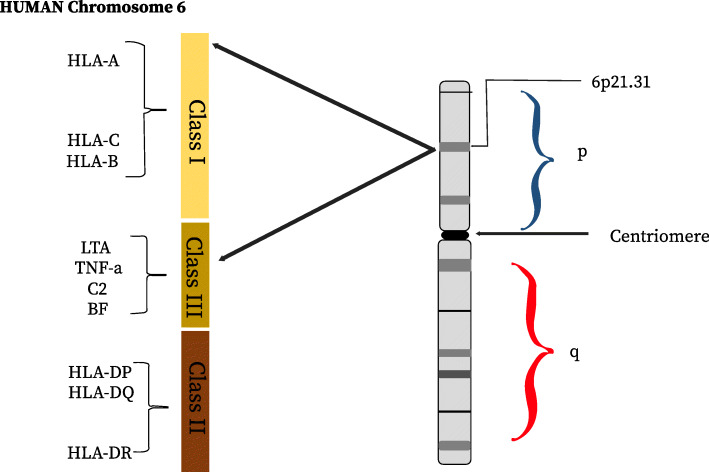
Schematic view of the HLA gene locus on the human chromosome 6, showing classical HLA class I (most common are HLA-A, HLA-B, and HLA-C) and class II (DR, DQ, DP, and DM) molecules in relation to other immune response gene found on the class III region

Human leukocyte antigen locus is a master regulator of immunity against infections. It potentially seems to be a crucial agent influencing the susceptibility and severity of COVID-19. Genetic variability across the three major histocompatibility complex (MHC) class I genes (human leukocyte antigen A [HLA-A], HLA-B, and HLA-C genes) could be seen to affect the susceptibility and severity of several infectious diseases. HLA molecules are encoded by a series of 21 protein-coding loci which lie among several other genes and pseudo genes at the 6p21 region of the chromosome 6 of the human genome (Fig. 2) [46]. These certain immune system genes, called human leukocyte antigen genes (HLA), are involved in recognizing pathogens, and they vary from one individual to another. These variations can influence how well the immune system recognizes a given pathogen. Their trans-membrane proteins which are encoded by the classical (A, B, C, DR, DQ, and DP) HLA genes (Fig. 2) are principally involved in the presentation of small pathogen-derived peptides to the T cells at the cell surface, which eventually triggers an immune response [20, 46]. The human leukocyte antigen (HLA) alleles are therefore a critical component of the viral antigen presentation pathway and have been shown in previously reported studies to cause differences in viral susceptibility and severity of diseases [7, 20]. Effective host response against viruses which evade the early innate defenses eventually relies heavily on HLA-restricted T cell responses through effective presentation of the viral epitopes by APCs (antigen-presenting cells) such as dendritic cells to CD8 T lymphocytes (CTLs) through class I HLA. The HLA class I antigen presentation leads to the clonal expansion of HLA-restricted CD8 cytotoxic T lymphocytes (CTL). The CTL response is essential to the intracellular antiviral defense [46, 47]. The advent of population-based biometrics approach to the study of infectious diseases in the 1950s gave rise to important results concerning viruses as related with these human leukocyte antigen (HLA) alleles. For instance, associations between HLA genotype and disease severity extend broadly to SARS-CoV-1, influenza virus, and several other unrelated viruses [29]. This result shows that some HLA class I alleles are strongly associated with lower viral loads in the blood and slower disease progression in individuals infected with human immunodeficiency virus (HIV), and homozygotes for a type III IFN (IFNL3-IFNL4) haplotype are more likely to clear hepatitis C virus spontaneously during primary infection. In some cases of human immunodeficiency virus 1 (HIV-1), HLA types HLA-A*0205 may have reduced risk of seroconversion. For dengue virus, HLA alleles HLA-A*0207 and HLA-B*51 are associated with increased secondary disease severity among a distinct ethnic group [29, 32]. This genetic variation among individuals may provide explanations for the differences in the various response of the immune system.

In an experiment performed by Lin et al. [31] during the 2003 Taiwan epidemic of SARS-CoV-1, HLA-class I and II allele typing by the use of PCR-SSOP was carried out on 37 probable cases of SARS, with 28 symptomatic fever patients later excluded as probable SARS, and 101 non-infected health care workers who were exposed or might have been possibly exposed to SARS-CoV-1 [31]. In addition, a control set of 190 normal healthy unrelated Taiwanese were also used in this analysis with a general view of screening for individuals at higher risk. The distribution of HLA class I and II alleles in both cases and control groups were examined for the presence of association to a genetic susceptibility or resistance to the SARS-CoV-1 infection. The result from this analysis of infected SARS patients and high risk health care workers groups showed HLA-B*4601 and HLA-B*5401 as the most probable elements that may be favoring SARS-CoV-1infection. After the selection and comparison of only “severe cases” patient group from the infected “probable SARS” patient group and with the high-risk health care workers group, HLA-B*4601 was shown to be significantly associated with the severity of SARS-CoV-1 [31]. The Taiwan indigenous peoples had considerably low reports of the SARS-CoV-1 probably because they have no HLA-B* 4601 but high frequency of HLA-B* 1301 instead which makes them genetically distinct from the Taiwanese general population who were more susceptible to severe cases of the infection. In a more recent research, Nguyen et al. [32] reported a comprehensive in silico analysis of the viral peptide-MHC class I binding affinity across HLA-A, HLA-B, and HLA-C genotypes for all SARS-CoV-2 peptides. This was carried out in order to discover how genetic variability across the three major histocompatibility complex (MHC) class I genes [HLA] A, B, and C may affect both severity and susceptibility to the SARS-CoV-2 infection. After SARS-CoV-2 proteome was successfully sampled and presented by a diversity of HLA alleles, analysis showed that HLA-B*4601 had the fewest predicted binding peptides for SARS-CoV-2, which suggests that people with this allele may be particularly vulnerable or even present severe conditions to COVID-19 [32]. This is very well correlated with the previous description shown above for SARS-CoV-1 [31]. It was also discovered that HLA-B*1503 showed the best capacity to present highly conserved SARS-CoV-2 peptides that are present among all other common human coronaviruses. This could also suggest a possibility to enable cross-protective T cell-based immunity [32].

Furthermore, the intracellular antiviral defense by CTLs is also complimented by CD4 T lymphocytes (TH cells) through class II HLA. In the humoral immune response, CD4 T lymphocytes augment the responses of CTL and provide help for the generation of specific antiviral antibodies [46, 47]. The correct activation of T helper cells (TH) for the correct production of antibodies by B cells and seroconversion is critically regulated by HLA class II genes; therefore, genetic variations for these molecules might constitute a varied humoral immune response to the clearance of SARS-CoV-2. Recently, Braun et al. [38] performed an experiment which focused on the S-specific CD4 T cell responses in 18 patients with mild, severe, or critical COVID-19 involving the use of overlapping peptide pools and induced CD154 and CD137 co-expression as a readout for antiviral CD4 T cells. Such cells were present in 83% of cases and presented with enhanced CD38, HLA-DR expression which showed recent in vivo activation. This study described the presence of activated CD4 T cells by the expression of HLA-DR [38]. In previous studies carried out on other viruses, observations were made to the associations between HLA alleles and hepatitis C virus (HCV) persistence as seen in HLA-DRB1*0301, or a relatively spontaneous clearance which is associated with HLA-A*0201, A*1101, B*5701, B*5703, C*0102, DQB1*0301, DRB1*0101, and DRB1*0401 [48–55] (Table 2). However, meta-analysis of HCV clearance stressed that these associations depend on the population origin [46].

HLA genes are also involved in the processes that culminate the composition of TCR repertoire by affecting both the intra- and extrathymic clonal selection. This partially accounts for the individual variations in the immune responses to pathogens [56]. For a novel pathogen like the SARS-CoV-2, a protective T cell-driven immune response to such unpredicted antigen would require the immune system to generate a new and diverse TCR repertoire [56]. It is possible that loss-of-function variation in some HLA genes may impair the ability to generate new and diverse TCR repertoire thereby leading to insufficient T lymphocyte response against the virus. In the case of naive TCR repertoire to SAR-CoV-2, thymopoiesis can generate SAR-CoV-2 reactive T cells. Moreover, memory TCR repertoire which are antigen-experienced T cells against coronaviruses have been reported to still generate responses against novel viruses such as the SARS-CoV-2 suggesting cross reactive T cell recognition between circulating common cold coronaviruses and SARS-CoV-2 [56, 57]. However, diversity of TCR repertoire has been described to decline with aging. The decrease in TCR diversity based on age was demonstrated in various high-throughput sequencing [TCR sequencing (TCR-seq)] technology studies. In a significant report, it was estimated that the TCRβ diversity in naïve T cell repertoires was 60–120 million for individuals in the early two decades of their life, whereas, a declined to about 8–57 million was observed in individuals who are over 70 years old [58]. Furthermore, the evidence of aging in TCR repertoire diversity is demonstrated in the antiviral response against the human influenza A virus [59, 60]. This may be of particular interest in the context of COVID-19, considering that the mortality of elderly patients with COVID-19 is higher than that of young and middle-aged patients [61–63]. Whether the aged and less diverse TCR repertoire impacts the ability to generate a sufficiently robust T cell response against SARS-CoV-2 in older patients still requires further studies.

## Genetic defects of in-born immunity and childhood mortality

Generally, global data shows a relatively low level of mortality among young patients with COVID-19 [64]. In the significant cases of severity observed in some few reports among this category of people, scientific efforts have been able to identify possible causes of such severity or death. Casanova et al. [29] and the COVID Human Genetic Effort were launched in a bid to facilitate this quest. Their hypothesis proposes that previously healthy, young patients with severe COVID-19 carry causal genetic variants which are responsible for differences in childhood mortality [6, 10]. Recently, results from these global COVID Human Genetic Effort revealed the essential roles for the double-stranded RNA sensor TLR3 and its regulation on type I IFN cell-intrinsic immunity in the control of SARS-CoV-2 infection. In the experiment to identify genetic defects associated with in-born errors of immunity at 8 of 13 candidate gene loci involved in the TLR3 and IRF7-dependent induction, Zhang et al. [37] discovered that at least 3.5% of patients with life threatening COVID-19 pneumonia had known autosomal recessive IRF7 and IFNAR1 deficiencies or autosomal dominant TLR3 and IRF3 deficiencies. Similarly, in the earlier case series of the 4 young men from 2 unrelated families with severe COVID-19, Caspar et al. [6] speculated that a unique loss-of-function variants in X-chromosomal TLR7 caused patients to require mechanical ventilation in the ICU with the death of one of the patients. Their analyses showed that the patients displayed impairment of transcriptional host type I IFN response. Furthermore, evidence has also recently shown the impaired upregulation expression of IRF and IFNB1. Bastard et al. [65] described that B cell auto-immune inborn errors of type I IFN immunity for patient’s whose adaptive auto-immunity impaired the innate and intrinsic antiviral immunity. These specific in-born errors of immunity provide insight to possible causes of mortality among relatively young individuals. It is also possible that mutations in some of these important genes along life may also serve as clue to understanding the outcome of severity in the much older patients. Discoveries are progressively indicating that genetic variants are critical for the outcome of not just SARs-CoV-2 but many other viral infections. However, in the case of the SARs-CoV-2 infection, further investigations are necessary among the young patients.

It is a herculean task to detect all the genetic factors that play important roles in the cause of the severity of a particular infection. The complexity of the interaction of these genetic factors within the human genome is yet to be fully known and understood. However, advances in genomics and DNA sequencing technologies in recent decades tend to find answers to this daunting question. In the case of the SARS-COV-2 virus, the rapid nature of mutation of the virus could be another factor of genetic consideration which stands different from the dynamics of the genetics of the human host. It is also possible that mutations of the single nucleotide polymorphism found along the HLA gene of individuals in conjunction with the ACE2 genes and several other toll-like receptor genes may be of notable significance for further research [33, 46].

## Conclusion

The understanding of the possible causes of the variation of severity of SARS-CoV-2 infection among patients is very important in identifying patients who are at a higher risk of the infection. This would lead to the development of technique that harnesses greater precautionary measures in a bid to protect such individuals from rapid exposure to SARS-CoV-2. Furthermore, should in case a vaccine against SARS-CoV-2 is discovered soon, individuals with high risk of the infection could be prioritized for the vaccination exercise. Identifying the genetic basis could also prove important in the discovery of resistant genes to SARS-CoV-2, which would provide pharmacological targets for preventing or reducing the viral infection in other individuals.

## Data Availability

There is no availability of data and materials.

## Abbreviations

ACE: Angiotensin-converting enzyme
ACE2: Angiotensin-converting enzyme 2
ARDS: Acute respiratory distress syndrome
COVID: Coronavirus disease
HLA: Human leucocyte antigen
HLH: Hemophagocytic lymphohistiocytosis
ICU: Intensive care unit
IFN: Interferon
IL: Interleukin
Mers-Cov: Middle East respiratory syndrome coronavirus
PAMP: Pathogen-associated molecular pattern
PRRs: Pattern recognition receptors
RAS: Renin-angiotensin system
SARS-Cov-2: Severe acute respiratory syndrome coronavirus 2
SNPs: Single nucleotide polymorphisms
TLR: Toll-like receptor
TMPRSS2: Trans-membrane serine protease 2
TNF: Tumor necrosis factor
